# *Bombyx mori* Silk Fibroin Regeneration in Solution of Lanthanide Ions: A Systematic Investigation

**DOI:** 10.3389/fbioe.2021.653033

**Published:** 2021-06-10

**Authors:** Giorgio Rizzo, Marco Lo Presti, Cinzia Giannini, Teresa Sibillano, Antonella Milella, Giulia Guidetti, Roberta Musio, Fiorenzo G. Omenetto, Gianluca M. Farinola

**Affiliations:** ^1^Dipartimento di Chimica, Università degli Studi di Bari “Aldo Moro”, Bari, Italy; ^2^Silklab, Department of Biomedical Engineering, Tufts University, Medford, MA, United States; ^3^CNR IC–Institute of Crystallography, Bari, Italy

**Keywords:** silk fibroin, lanthanide doped fibroin, silk fibroin dissolution, silk fibroin recovery, biomaterials

## Abstract

Silk Fibroin (SF) obtained from *Bombyx mori* is a very attractive biopolymer that can be useful for many technological applications, from optoelectronics and photonics to biomedicine. It can be processed from aqueous solutions to obtain many scaffolds. SF dissolution is possible only with the mediation of chaotropic salts that disrupt the secondary structure of the protein. As a consequence, recovered materials have disordered structures. In a previous paper, it was shown that, by modifying the standard Ajisawa’s method by using a lanthanide salt, CeCl_3_, as the chaotropic agent, it is possible to regenerate SF as a fibrous material with a very ordered structure, similar to that of the pristine fiber, and doped with Ce^+3^ ions. Since SF exhibits a moderate fluorescence which can be enhanced by the incorporation of organic molecules, ions and nanoparticles, the possibility of doping it with lanthanide ions could be an appealing approach for the development of new photonic systems. Here, a systematic investigation of the behavior of degummed SF in the presence of all lanthanide ions, Ln^+3^, is reported. It has been found that all lanthanide chlorides are chaotropic salts for solubilizing SF. Ln^+3^ ions at the beginning and the end of the series (La^+3^, Pr^+3^, Er^+3^, Tm^+3^, Yb^+3^, Lu^+3^) favor the reprecipitation of fibrous SF as already found for Ce^+3^. In most cases, the obtained fiber preserves the morphological and structural features of the pristine SF. With the exception of SF treated with La^+3^, Tm^+3^, and Lu^+3^, for all the fibers re-precipitated a concentration of Ln^+3^ between 0.2 and 0.4% at was measured, comparable to that measured for Ce^+3^-doped SF.

## Introduction

Silk fibroin (SF) is the major component of the *Bombyx mori* cocoons (70% ca) and readily available in nature. Its peculiar structural, mechanical and biological properties make it a very attractive biopolymer (Omenetto and Kaplan, 2010; Ling et al., 2018) which offers unlimited opportunities for functionalization, processing and biological modifications (Rockwood et al., 2011; Reddy, 2020). Due to its biocompatibility and biodegradability, SF is an excellent biomaterial for a wide range of biomedical applications, for example drug delivery, tissue engineering, including cartilage and bone, skin tissue, ligaments, cornea, tympanic membrane, and implantable devices such as artificial kidney, and vascular grafts wound dressings. (Lawrence et al., 2008; Kundu et al., 2013; Koh et al., 2015; Melke et al., 2016). It can be processed from aqueous solutions and converted into a variety of versatile systems (Qi et al., 2017; Huang et al., 2018; Guo et al., 2020), such as hydrogels sponges, foams, solid matrices, ultra-resistant, optically transparent and flexible films among others. Moreover, doping with various organic and inorganic dopants opens up the way to a wide variety of applications ranging from supported catalysts (Rizzo et al., 2020a) to environmentally sustainable devices for photonics, electronics, and optoelectronics (Tao et al., 2012; Zhu et al., 2016). In fact, SF exhibits a moderate fluorescence (it can be excited at 277 nm and emits light at 345 nm) (Georgakoudi et al., 2007) that has been ascribed to the aromatic moieties of Tryptophan and Tyrosine, commonly used, together with Phenylalanine, as fluorescent probes to study the structure of proteins. SF fluorescence can be enhanced by the incorporation of organic molecules, ions and nanoparticles within the fibers, and the possibility of doping with lanthanide (Ln) ions opens new possibilities for photonic systems (Lee et al., 2020).

The use of Ln-doped fibers in photonics is well-known, and several studies have reported that the presence of lanthanides can confer photochromic, luminescent and fluorescent properties as well as up-conversion ability to fibers. Examples could be Eu^+3^ or Dy^+3^ strontium aluminate luminescent fibers produced by a special spinning technology, with the substrate of polyester, nylon, or polypropylene resin (Guo et al., 2011), fluorescent Pr^+3^ and Dy^+3^-doped Chalcogenide fibers (Chahal et al., 2016), Er^+3^/Yb^+3^/Tm^+3^ tri-doped tellurite glass microsphere coupled by tapered fibers (Liu et al., 2020), fluorescent silks obtained from silkworms fed with rare-earth upconverting phosphors (Zheng et al., 2018). Moreover, Ln-doped fibers have been employed in the production of lasers (amplifiers rare-earth-doped silica and fluorozirconate fibers, particularly erbium-doped fibers) (Digonnet, 2001) and smart Dy^+3^ and Eu^+3^ doped stimuli-responsive textiles (Shen et al., 2021). In addition, Tryptophan and Tyrosine can act as sensitizers for lanthanide ions enhancing their emission (da Rocha et al., 2020). Therefore, combining the mechanical and optical properties of SF with those of lanthanides ions could be an interesting approach for the development of new photonic systems, particularly appealing due to the high biocompatibility and biodegradability of SF.

Few examples of SF films and powders doped with lanthanides have been reported so far and are limited to Eu^+3^ and Tb^+3^ (Pugina et al., 2019a,b; da Rocha et al., 2020). In a previous work (Rizzo et al., 2020b) we have described a simple method for doping fibrous SF with Ce^+3^. In particular, in the framework of a systematic study on the effects of changing the chaotropic salt in Ajisawa’s solvent (CaCl_2_ in H_2_O/EtOH in a molar ratio 1:8:2), used as standard protocol to solubilize SF (Ajisawa, 1998), we found that when replacing CaCl_2_ with the hydrated lanthanide salt CeCl_3_⋅7H_2_O while maintaining all the other conditions unchanged, not only SF was completely dissolved, but it reprecipitated in a fibrous form very quickly and spontaneously during the dialysis purification. The structural characterization of the resulting material demonstrated that the fibers obtained preserved most of the morphological and structural properties of the original degummed SF (Marsh et al., 1955; Guo et al., 2018), with a highly ordered molecular organization, and that they were doped with Ce^+3^ ions, in a concentration of about 0.3 wt%, distributed both within its amorphous regions and the β-crystalline domains.

This protocol represents the first method to obtain SF in a fibrous form after solubilization (the fibroin is normally re-obtained as aqueous solutions or gels). In addition, the SF obtained is doped with Ce^+3^ ions. In principle, this simple process could be extended to other lanthanide ions, possibly resulting in a more generally applicable protocol to produce new silk-based doped materials.

Here we report the results of a systematic investigation of the behavior of degummed SF solution in the presence of all lanthanide ions in the Periodic Table, from Lanthanum to Lutetium, in the form of chloride salts, with the only exclusion of Promethium, due to its radioactivity. The main goals of our paper are: (i) disclosing a new protocol to obtain doped fibroin in fibrous form after solubilization in the presence of Ln^+3^ ions and (ii) characterizing structural and morphological properties of the fibrous materials obtained.

## Materials and Methods

All hydrated lanthanide chlorides were purchased from NOVA ELEMENTS SAS (Palermo, Italy). All other reagents and solvents were purchased from Sigma Aldrich and were used without any further purification.

### Preparation of SF Samples

#### Degummed SF

Degummed SF was obtained from *Bombyx mori* cocoons purchased from Tajima Shoji (Japan), following previously described procedures (Rockwood et al., 2011). To remove sericin, the cocoons were cut into fourths, shredded and boiled for 30 min in an aqueous solution of Na_2_CO_3_ 0.02 M, then rinsed thoroughly with bidistilled water to remove the residual sericin and the excess of salt. The fibers were dried at ambient conditions for 24 h.

#### Dissolution of Degummed SF With Different Lanthanide Salts by modified Ajisawa’s Method

The standard Ajisawa’s method (Ajisawa, 1998) was applied to dissolve degummed SF, using hydrated lanthanide chlorides (LaCl_3_⋅7H_2_O, CeCl_3_⋅7H_2_O, PrCl_3_⋅7H_2_O, NdCl_3_⋅6H_2_O, SmCl_3_⋅6H_2_O, EuCl_3_⋅6H_2_O, GdCl_3_⋅6H_2_O, TbCl_3_⋅6H_2_O, DyCl_3_⋅6H_2_O, HoCl_3_⋅6H_2_O, ErCl_3_⋅6H_2_O, TmCl_3_⋅6H_2_O, YbCl_3_⋅6H_2_O, LuCl_3_⋅6H_2_O) as chaotropic agents instead of CaCl_2_. The same molar ratios of the standard Ajisawa’s protocol were used (0.528 g of CaCl_2_, corresponding to 4.76 mmol, 0.69g of H_2_O and 0.44g of EtOH, molar ratio CaCl_2_: H_2_O: EtOH 1:8:2). Hence, for all the lanthanide chlorides 0.125 g of degummed SF were added to a solution prepared by dissolving 4.76 mmol of the salt (see Supplementary Table 1) in 0.69g of H_2_O and 0.44g of EtOH. The mixtures were heated at 60°C for 4 h. The obtained solutions were dialyzed with bidistilled water, using a regenerated cellulose dialysis membrane (MWCO 12.400 g mol^–1^) to remove salt and ethanol. In the presence of La^+3^, Ce^+3^, Pr^+3^, Er^+3^, Tm^+3^, Yb^+3^, Lu^+3^, and partially for Dy^+3^, SF reprecipitated within the dialysis cassette after 4 h and the dialysis purification step was stopped after 12 h, changing water every 4 h. The Ln^+3^/SF fibers were rinsed and washed with bidistilled water several times and finally air dried at room temperature. The amount of SF recovered was determined by weighting methods.

For Nd^+3^, Sm^+3^, Eu^+3^, Gd^+3^, Tb^+3^, and partially for Dy^+3^, clear SF aqueous solutions were obtained after dialysis and hence stored a 4°C. From these solutions, drop-casted films were obtained for further investigations.

After dialysis of SF solutions obtained with Ho^+3^ a hydrogel was obtained.

Different concentrations of CeCl_3_ in the range 1.20–8.40 mmol gsolvent^–1^were tested, but SF was completely dissolved only when the same molar ratio of the Ajisawa’s protocol was used (Rizzo et al., 2020b). The same results were obtained for LaCl_3_.

### Methods for Structural Characterization

The regenerated SF fibers recovered after treatment with La^+3^, Pr^+3^, Er^+3^, Tm^+3^, Yb^+3^, and Lu^+3^ were characterized by scanning electron microscopy (SEM), Wide-Angle X-ray Scattering (WAXS), birefringence, Attenuated Total Reflectance Fourier Transformed Infrared Spectroscopy (ATR-FTIR) and X-Ray Photoelectron Spectroscopy (XPS). Further, drop-casted films from SF solutions obtained by Nd^+3^, Sm^+3^, Eu^+3^, Gd^+3^, and Te^+3^ treatment were characterized by ATR-FTIR.

#### SEM Characterization

SEM analysis was carried out by a VP Field emission SEM EDS Zeiss Sigma 300 equipped with an in lens backscattered and secondary electron detectors. An accelerating voltage of 15 kV was used and the working distance was set to 6.5 mm. FE-SEM samples were placed onto stainless-steel sample holders with carbon tape. A carbon sputtering was performed onto samples before analyses in order to prevent electron charging due to the low sample conductivity, thus enhancing topography imaging. The EDS detector was an X-Max-Silicon Drift Detector (SDD)–Nano analysis–Oxford Instruments. The acquired images were analyzed with AZtec Software.

#### XPS Characterization

The surface chemical composition of regenerated SF loaded with lanthanide rare earths was investigated by X-ray Photoelectron Spectroscopy (XPS). Analyses were performed with a Scanning XPS Microprobe (PHI 5000 Versa Probe II, *Physical Electronics*), equipped with a monochromatic Al K_α_ X-ray source (1486.6 eV) operated at 15 kV with a spot of 100 μm and a power of 24.8 W. Survey (0–1,200 eV) and high-resolution spectra (C1s, O1s, N1s, Lu4f, Tm4d, Pr3d, Dy4d, Yb4d, Er4d, La3d) were recorded in FAT (Fixed Analyzer Transmission) mode at a pass energy of 117.40 and 29.35 eV, respectively. In the set conditions, the analyzer energy resolution (FWHM, full width at half-maximum height), measured on the silver Ag 3d_5/2_ photoemission line, was 0.7 eV for a pass energy of 29.35 eV. All spectra were acquired at a take-off angle of 45° with respect to the sample surface. Surface charging was compensated using a dual beam charge neutralization, with a flux of low energy electrons (∼1 eV) combined with very low energy positive Ar ions (10 eV). The hydrocarbon component of C1s spectrum was used as the internal standard for charging correction and it was fixed at 285.0 eV (Moulder et al., 1992). Scanning X-ray beam induced secondary electron images were acquired to finely select analysis areas. Furthermore, on each sample, three different spots were analyzed for statistical purposes. MultiPak (*Physical Electronics*) software was used to process acquired spectra.

#### WAXS Characterization

WAXS experiments were performed at the X-ray Micro Imaging Laboratory (XMI-LAB) of the Institute of Crystallography of CNR-Bari (Altamura et al., 2012; Sibillano et al., 2016; Siliqi et al., 2016). The laboratory is equipped with a Fr-E + SuperBright rotating copper anode microsource (λ = 0.154 nm, 2,475 W) coupled through a focusing multilayer optics Confocal Max-Flux to a SAXS/WAXS three pinholes camera equipped for X-ray scanning microscopy. An image plate (IP) detector (250 × 160 mm^2^, with 100 μm effective pixel size), with an off-line RAXIA reader, was used to collect WAXS data. The spot size at the sample position was around 200 μm. The detector was placed at around 10 cm from the samples, giving access to a range of scattering vector moduli (q = 4πsinϑ/λ) from 0.3 to around 3.5 Å^–1^, which corresponds to 1.8–25 Å d-spacing range.

#### Birefringence Characterization

A customized Olympus Inverted IX71 microscope equipped with a DSLR (digital single-lens reflex) camera (Canon Rebel EOS-SL1) and with a halogen lamp (Olympus, U-LH100L-3) as light source was used to perform optical microscopy. Bright-field transmission micrographs between crossed polarizers were collected using a 20× objective (Olympus, LUCPlanFL N, NA 0.45). For each Ln^+3^ treatment individual fibers were analyzed and the most representative micrograph was reported.

#### ATR-FTIR Characterization

ATR-FTIR spectra were acquired with a Perkin Elmer Spectrum Two Spectrophotometer equipped with a 2 × 2 mm Diamond crystal. Spectra were recorded in the range 4,000–400 cm^–1^ with a 2 cm^–1^ resolution, using 0.25 cm^–1^ acquisition interval and acquiring 32 scans for each sample.

## Results

According to Ajisawa’s standard method (Ajisawa, 1998), hydrated LnCl_3_ in H_2_O/EtOH (in a molar ratio 1:8:2), were used to dissolve degummed SF. The mixtures were heated at 60°C for 4 h. The resulting SF solutions were dialyzed against bidistilled water to remove the residual salt and ethanol. Regenerated fibers were rinsed several times with water and let air-dry. Solutions and hydrogel were stored at 4°C.

**FIGURE 1:**
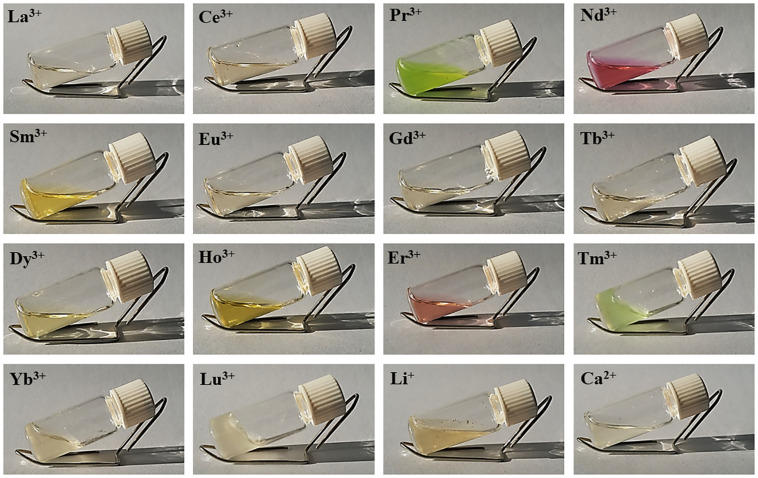
Solutions of degummed SF dissolved in hydrated LnCl_3_/H_2_O/EtOH (1:8:2). The picture of SF dissolved in LiBr 9M in H_2_O and CaCl_2_/H_2_O/EtOH (1:8:2) (standard Ajisawa’s solvent) is also reported.

All lanthanides chlorides in H_2_O/EtOH were able to dissolve degummed SF (Figure 1). As already found for CeCl_3_⋅7H_2_O, when using hydrated LaCl_3_, PrCl_3_, ErCl_3_, TmCl_3_, YbCl_3_, and LuCl_3_ as chaotropic salts, a fibrous material reprecipitated within the dialysis cassette (Table 1). In all these cases the recovery of the fiber was almost quantitative. With DyCl_3_ the recovery of the fiber was only partial (48–51%) whilst with HoCl_3_ a hydrogel was obtained. Intermediate lanthanides of the series (NdCl_3_, SmCl_3_, EuCl_3_, GdCl_3_, and TbCl_3_) did not induce precipitation of a regenerated fibrous material leading to, at the end of the purification process, clear and stable solutions that could be used to obtain films and/or other scaffolds.

**TABLE 1:** Lanthanide salts used for SF dissolution/regeneration protocol. For comparison, calcium chloride standard salt is listed in the last row.

**Lanthanide**	**Salt**	**Ln^+3^ Ionic radius (Å) (Seaborg, 1978)**	**Dialysis result**
Lanthanum	LaCl_3_⋅7H_2_O	1.061	Fibers
Cerium	CeCl_3_⋅7H_2_O	1.034	Fibers
Praseodymium	PrCl_3_⋅7H_2_O	1.013	Fibers
Neodymium	NdCl_3_⋅6H_2_O	0.995	Solution
Samarium	SmCl_3_⋅6H_2_O	0.964	Solution
Europium	EuCl_3_⋅6H_2_O	0.950	Solution
Gadolinium	GdCl_3_⋅6H_2_O	0.938	Solution
Terbium	TbCl_3_⋅6H_2_O	0.923	Solution
Dysprosium	DyCl_3_⋅6H_2_O	0.908	Solution/Fiber
Holmium	HoCl_3_⋅6H_2_O	0.894	Gel
Erbium	ErCl_3_⋅6H_2_O	0.881	Fibers
Thulium	TmCl_3_⋅6H_2_O	0.869	Fibers
Ytterbium	YbCl_3_⋅6H_2_O	0.858	Fibers
Lutetium	LuCl_3_⋅6H_2_O	0.848	Fibers
Calcium	CaCl_2_	1.00	Solution

The structural and morphological properties of the solid fibers recovered after treatment with La^+3^, Pr^+3^, Er^+3^, Tm^+3^, Yb^+3^, and Lu^+3^ salts (hereafter indicated in the text as Ln^+3^/SF) in H_2_O/EtOH were analyzed. Further, drop-casted films from SF solutions obtained by Nd^+3^, Sm^+3^, Eu^+3^, Gd^+3^, and Te^+3^ solution treatment were characterized by ATR-FTIR. All these experiments showed that the material recovered is, in fact, SF.

### Morphology of Ln^+3^/SF Fibers by SEM

The morphological features of the solid SF fibers recovered after treatment with lanthanides were studied by SEM.

**FIGURE 2:**
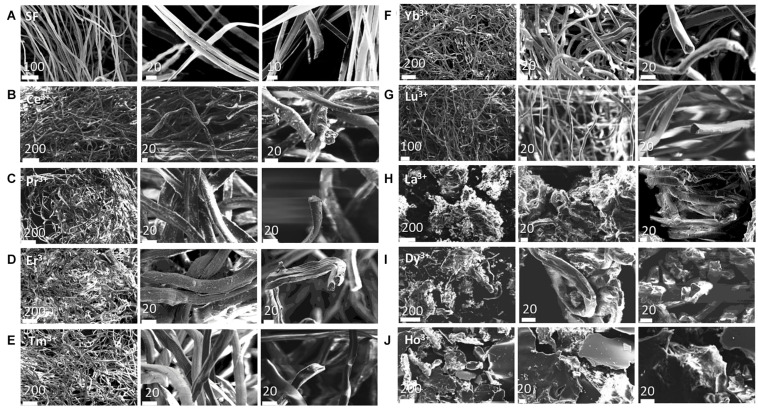
SEM images of the Analyzed samples at two scale bars in μm (indicated on each figure) with the cross-sectional views. **(A)** degummed SF; **(B)** Ce^+3^/SF; **(C)** Pr^+3^/SF; **(D)** Er^+3^/SF; **(E)** Tm^+3^/SF; **(F)** Yb^+3^/SF; **(G)** Lu^+3^/SF; **(H)** La^+3^/SF; **(I)** Dy^+3^/SF; **(J)** Ho^+3^/SF.

The SEM images are reported in Figures 2C–J (larger images are shown in Supplementary Figure 1 of the Supporting Information) and can be compared with those obtained for degummed SF (Figure 2A; Shen et al., 1998; Rizzo et al., 2020b) and SF doped with Ce^+3^ (Figure 2B), already reported in our previous paper (Rizzo et al., 2020b). The cross-sectional views of the fibers are also reported.

As reported in the literature (Shen et al., 1998; Rizzo et al., 2020b), the fibers of degummed SF (Figure 2A) are uniform, with a very smooth surface and they are organized in single brins with typical hemicylindrical cross-sections and an average diameter of 9.9 ± 0.5 μm. The SEM images of Pr^+3^/SF, Er^+3^/SF, Tm^+3^/SF, Yb^+3^/SF, and Lu^+3^/SF (Figures 2C–G) are comparable to those of Ce^+3^/SF: these materials have a fibrous microstructure and retains the fiber integrity of SF, but with reduced smoothness and straightness. The typical hemicylindrical shape of the fiber cross section is also retained, but, with the exception of Lu^+3^/SF, whose average diameter (11.2 ± 1.8 μm) is similar to that of degummed SF (9.9 ± 0.5 μm), all other lanthanide/fibers are characterized by larger cross-sections. The diameters of Ln^+3^/SF fibers, reported in Table 2, were calculated as the average of the dimensions of at least 30 fibers, measured directly on the SEM images recorded.

**TABLE 2:** Diameters (in μm) of degummed SF and Ln^+3^/SF fibers.

	**SF**	**Ce^+3^/SF**	**Pr^+3^/SF**	**Er^+3^/SF**	**Tm^+3^/SF**	**Yb^+3^/SF**	**Lu^+3^/SF**
Diameter	9.9 ± 0.5	20.4 ± 1.1	16.7 ± 0.5	19.4 ± 2.2	14.9 ± 1.8	17.5 ± 0.8	11.2 ± 1.8

La^+3^/SF and Dy^+3^/SF samples (Figures 2H,I) are constituted by small and disordered fragments of SF fibers, with very coarse surfaces and irregular cross-sections.

In addition, SEM analysis of the air-dried hydrogel obtained after treatment of SF with Ho^+3^ (Figure 2J) clearly shows a highly disordered structure with no evidence of a fibrillar structure.

### Inclusion of Ln^+3^ Ions Within SF by XPS

XPS investigation was performed to assess if the regenerated SF obtained after reprecipitation from LnCl_3_/H_2_O/EtOH solutions are contaminated by lanthanide ions. In fact, the low-resolution spectra of all regenerated fibers show photoelectron peaks originated from the lanthanide ions.

**FIGURE 3:**
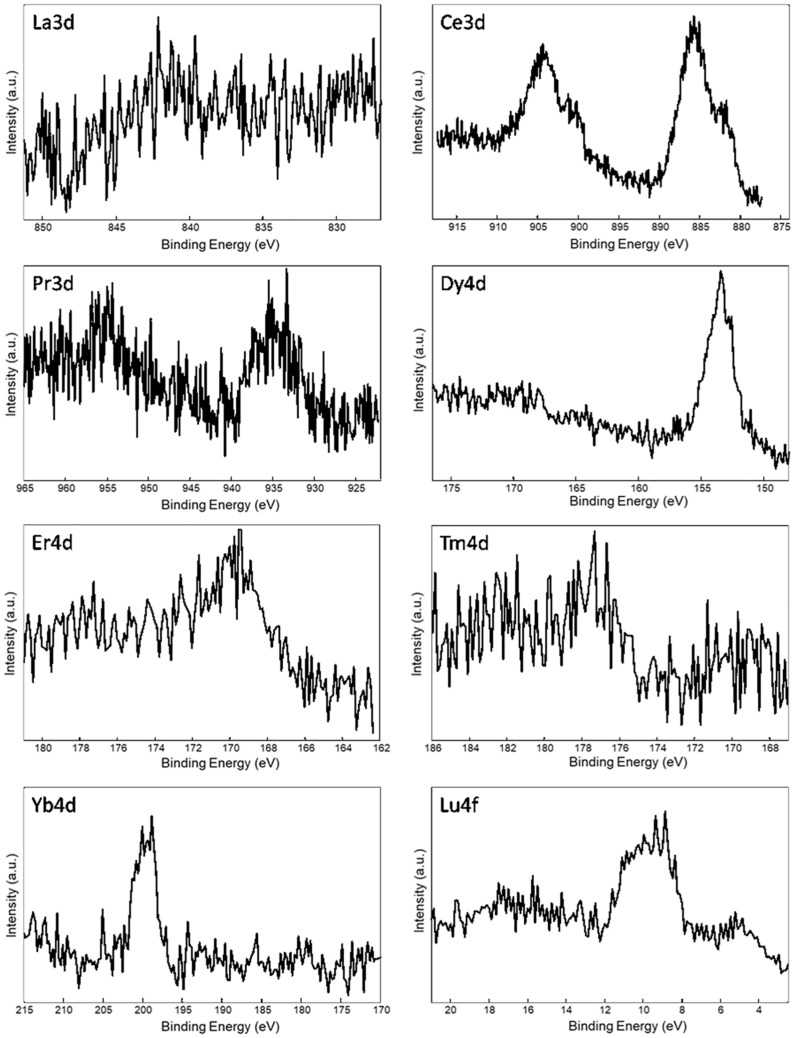
XPS spectra of Ln^+3^/SF regenerated fibers. Selected atomic orbital for metal investigation is indicated on each spectrum.

Table 3 reports the surface elemental content of Ln^+3^ ions for La^+3^/SF, Pr^+3^/SF, Dy^+3^/SF, Er^+3^/SF, Tm^+3^/SF, Yb^+3^/SF, and Lu^+3^/SF regenerated fibers. High resolution spectra of Ln^+3^ are shown in Figure 3. Ce^+3^ spectrum of Ce^+3^/SF is reported for comparison. The quantitative elemental composition of the fibers was also determined by high-resolution C1s, N1s, and Ln (La3d, Ce3d, Pr3d, Dy4d, Er4d, Tm4d, Yb4d, Lu4f) spectra (from peak areas, using tabulated sensitivity factors and spectrometer transmission function) as reported in the Supporting Information (Supplementary Table 2).

**TABLE 3:** XPS peak analyses of Ln^+3^/SF fibers.

**Sample**	**Peak**	**BE (eV)**	**at%**
Ce^+3^/SF	Ce3d	885.9	0.3
La^+3^/SF	La3d	837.4	<0.1
Pr^+3^/SF	Pr3d	933.7	0.4
Dy^+3^/SF	Dy4d	153.2	0.3
Er^+3^/SF	Er4d	168.9	0.2
Tm^+3^/SF	Tm4d	177.2	<0.1
Yb^+3^/SF	Yb4d	199.3	0.3
Lu^+3^/SF	Lu4f	8.4	<0.1

Ln^+3^ concentration ranges from less than 0.1 at%, which is the detection limit of the technique, to 0.4 at%.

These experimental data suggest that, besides Ce^+3^, only Pr^+3^, Dy^+3^, Er^+3^, and Yb^+3^ are detectable as contaminants of the SF. XPS spectra in Figure 3 do not allow to distinguish if the ions are included within the fiber during reprecipitation or are present only as impurities, probably coordinated to the fiber surfaces.

In order to get deeper insights into the chemical environment of lanthanide ions in the regenerated fibers, XPS spectra of DyCl_3_⋅6H_2_O and YbCl_3_⋅6H_2_O salts were acquired. In fact, as previously demonstrated for Ce^+3^/SF (Rizzo et al., 2020b), a shift toward lower binding energy (−1.3 eV) in the Ce3d spectrum of Ce^+3^/SF compared to the spectrum of CeCl_3_⋅7H_2_O suggested the existence of interactions between Ce^+3^ ions and the oxygen atoms of fibroin aminoacids within the fiber. The same comparison was performed herein for Dy^+3^ and Yb^+3^, which are those with higher concentration within the fibers and show well defined XPS spectra. For both the elements, no chemical shift was observed between the Ln^+3^ spectra as the salt and in the fibers. Therefore, differently from Ce^+3^, no evidence of a coordinating effect of the Ln^+3^ ions to SF was observed.

### Solid State Structure of Ln^+3^/SF by WAXS

**FIGURE 4:**
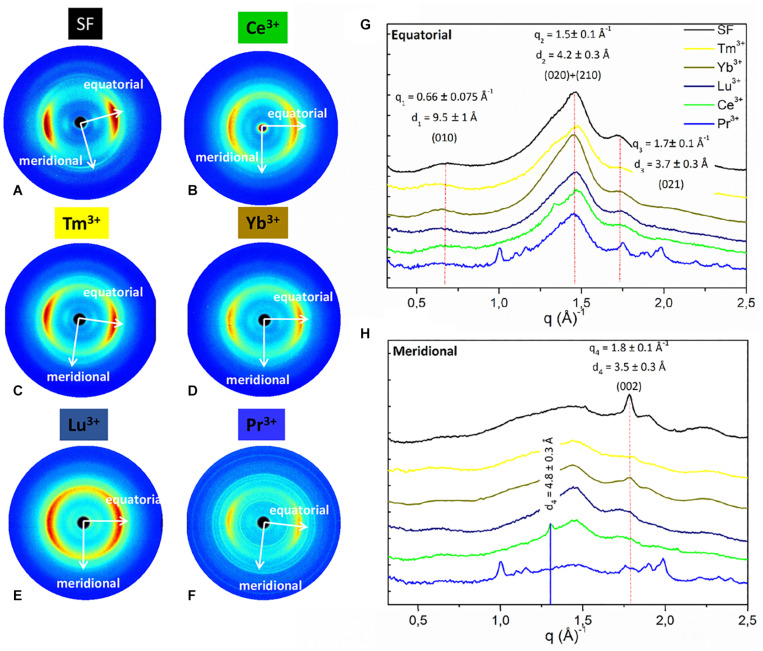
2D WAXS patterns of **(A)** degummed SF and regenerated fibers obtained after treatment with **(B)** Ce^+3^ **(C)** Tm^+3^; **(D)** Y^+3^; **(E)** Lu^+3^; **(F)** Pr^+3^, respectively, corresponding 1D **(G)** equatorial patterns and **(H)** meridional patterns. Vertical bars highlight the equatorial reflections at q_1_ = 0.66 ± 0.075 Å^− 1^ (d_1_ = 9.5 ± 1 Å), q_2_ = 1.5 ± 0.1 Å^− 1^ (d_2_ = 4.2 ± 0.3 Å), and q_3_ = 1.7 ± 0.1 Å^− 1^ (d_3_ = 3.7 ± 0.3 Å) and the meridional reflection at q_4_ = 1.8 ± 0.1 Å^− 1^ (d_4_ = 3.5 ± 0.2 Å) shifted at q_4_ = 1.3 ± 0.1 Å^− 1^ (d_4_ = 4.8 ± 0.2 Å) only for Ce^+3^. The equatorial and meridional peaks were indexed as follows: q_1_ the (010) reflection; q_2_ an overlap of the (020) and (210) reflections; q_3_ the (021) reflection; q_4_ the (002) reflection.

**FIGURE 5:**
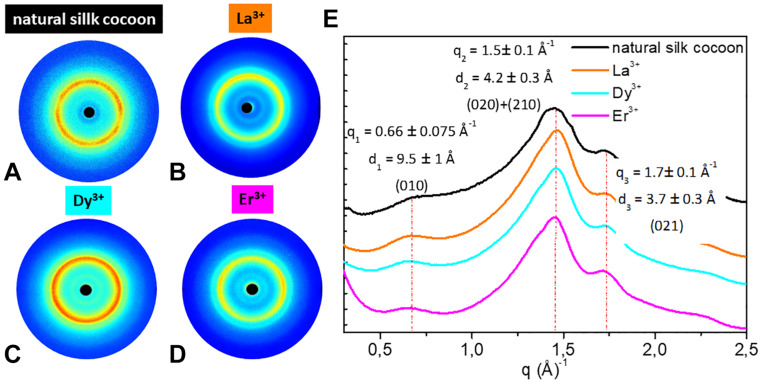
2D WAXS patterns of **(A)** natural silk cocoon and regenerated fibers obtained after treatment with **(B)** La^+3^; **(C)** Dy^+3^; **(D)** Er^+3^ respectively; **(E)** corresponding 1D WAXS patterns with vertical bars highlighting the reflections at q_1_ = 0.66 ± 0.075 Å^−1^ (d_1_ = 9.5 ± 1 Å), q_2_ = 1.5 ± 0.1 Å^−1^ (d_2_ = 4.2 ± 0.3 Å), and q_3_ = 1.7 ± 0.1 Å^−1^ (d_3_ = 3.7 ± 0.3 Å). These peaks were indexed as follows: q_1_ the (010) reflection; q_2_ an overlap of the (020) and (210) reflections; q_3_ the (021) reflection.

WAXS experiments were performed on the solid fibers regenerated after La^+3^, Dy^+3^, Er^+3^, Tm^+3^, Yb^+3^, Lu^+3^, and Pr^+3^ treatment. The 2D WAXS patterns (Figures 4B–F, 5B–D), once centered, calibrated and folded into 1D profiles, were compared with the WAXS patterns of the degummed SF and of *B. mori* cocoons (natural silk) (Figures 4A, 5A, respectively), (Shen et al., 1998; Guo et al., 2018; Rizzo et al., 2020b).

As previously reported (Rizzo et al., 2020b), within the same explored area of about 200 μm, degummed SF exhibits a typical cross β-diffraction signal (Figure 4A) whose intensity is anisotropically distributed along two main orthogonal directions labeled as meridional, along the fiber axis, and equatorial, perpendicular to the fiber axis (Guo et al., 2018). The 1D WAXS profile was integrated around both the equatorial and the meridional directions (black curves in Figures 4G,H, respectively). The 2D/1D data were indexed as the fiber diffraction pattern of the *Bombyx mori* silk II structure (Fossey et al., 1991; Guo et al., 2018), which has an orthorhombic structure with unit cell dimensions a = 9.68 ± 0.20 Å, b = 9.36 ± 0.18 Å, and c = 7.02 ± 0.14 Å. *B. mori* cocoons (natural silk) does not show a WAXS pattern with clear preferential orientation but rings with uniform intensity along the azimuth (Figure 5A). Its 1D WAXS profile was integrated along all the azimuth and is reported in Figure 5E (black curve).

SF regenerated after treatment with Ln^+3^ ions showed two typical structural behaviors:

(i) samples treated with Tm^+3^, Yb^+3^, Lu^+3^, and Pr^+3^ exhibit a 2D typical cross β-diffraction pattern (Figures 4C–F), similar to that of the degummed SF and Ce^+3^/SF, thus indicating a partial recovery of their fibrillar nature after reprecipitation.

(ii) Samples with the addition of La^+3^, Dy^+3^, and Er^+3^ show 2D WAXS patterns similar to that of the natural silk cocoon, i.e., without any preferential orientation (Figures 5B–D). The relevant 1D WAXS profiles are shown in Figure 5E (orange for La^+3^, cyan for Dy^+3^ and magenta for Er^+3^).

The d-spacings and corresponding *q*-values for all reflections are reported in Supplementary Table 2 of the Supporting Information. In details, the equatorial peaks at q_1_ = 0.66 ± 0.075 Å^–1^ (d_1_ = 9.5 ± 1 Å), q_2_ = 1.5 ± 0.1 Å^–1^ (d_2_ = 4.2 ± 0.3 Å), q_3_ = 1.7 ± 0.1 Å^–1^ (d_3_ = 3.7 ± 0.3 Å) were indexed as the (010), the overlap of the (020) and (210) reflections, and the (021) reflection, respectively. The latter forms an angle of about 30° with the equatorial direction (Supplementary Figures 2, 3 in the Supporting Information). The meridional peak at q_4_ = 1.8 ± 0.1Å^–1^ (d_4_ = 3.5 ± 0.2 Å) (Figure 5E), indexed as the (002) reflection, indicates the β-strands distance along the fiber axis, which yields to the c = 7.0 Å axial repetition and contains two peptide units. In our previous work (Rizzo et al., 2020b), the SF sample doped with Ce^+3^ ions showed a shift of the meridional (002) reflection from q_4_ = 1.8 Å^–1^ (d_4_ = 4.8 ± 0.2 Å) to q_4_ = 1.3 Å^–1^ (d_4_ = 4.8 ± 0.2 Å) (blue vertical bar in Figure 4H). This shift corresponded to an increase in the β-strands distance from 3.5 Å, in degummed SF, to 4.8 Å for Ce^+3^/SF and was ascribed to the incorporation of Ce^+3^ ions along the c-axis of the fiber. None of the Ln^+3^/SF samples show such a shift of the meridional peak position. Therefore, there is no sign of inclusion of La^+3^, Dy^+3^, Er^+3^, Tm^+3^, Yb^+3^, Lu^+3^, and Pr^+3^ ions within the crystalline domains of the fiber.

Finally, WAXS of the Pr^+3^/SF (Figure 4F) shows several additional sharp rings which are due to contamination.

### Birefringence

The results of WAXS characterization are supported by birefringence measurements. For optically anisotropic fibers, birefringence can be a useful tool in structural characterization since it can give information on the orientation of crystalline regions. In fact, birefringence colors arise from the interaction of incident polarized light with the anisotropic crystalline domains of the fibers with a preferred axial orientation, i.e., β-sheet nanocrystal in SF aligned along the fiber axis (Founda and El-Tonsy, 1990; Viney et al., 1993). The effect of the lanthanides on the silk fiber birefringence can be monitored by brightfield transmission microscopy between crossed polarizers.

**FIGURE 6:**
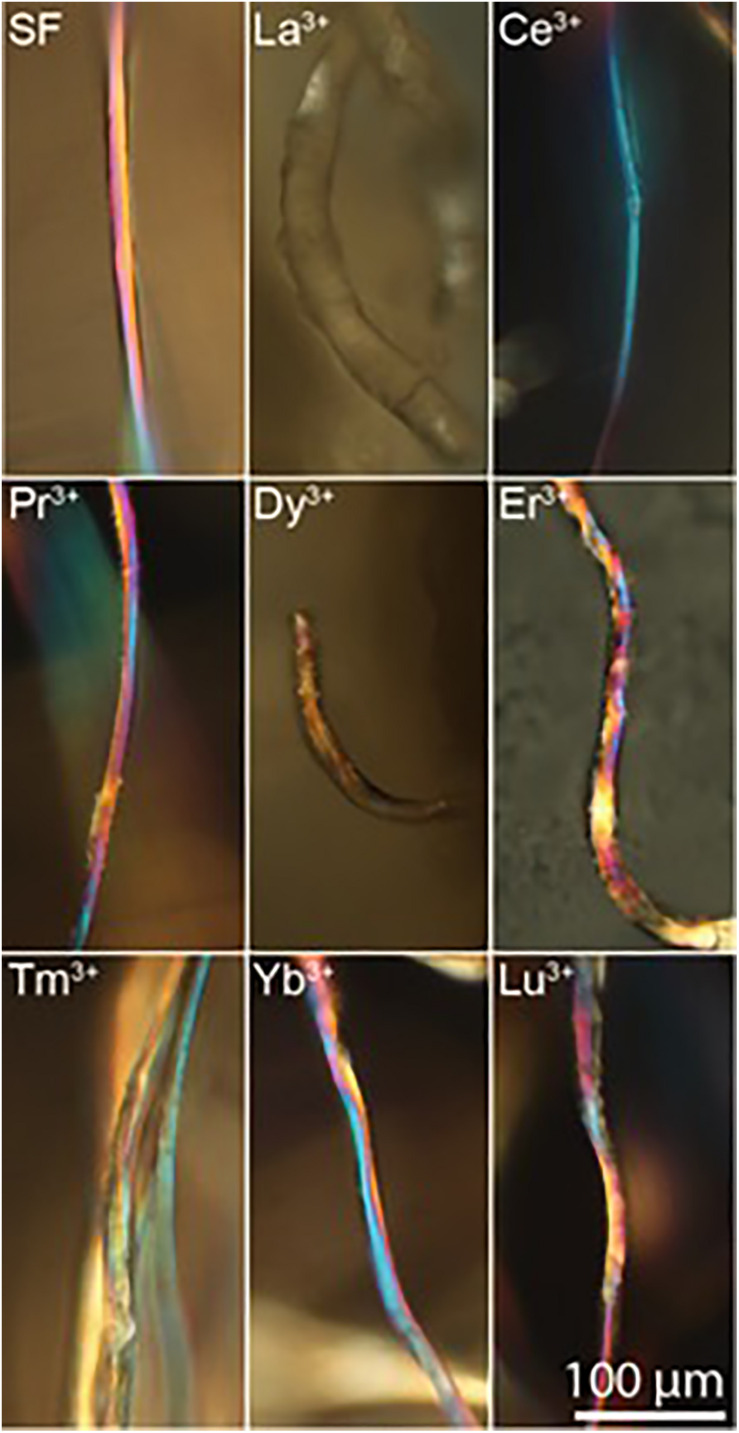
Birefringence images of regenerated Ln^+3^/SF fibers (degummed SF, La^+3^/SF, Ce^+3^/SF, Pr^+3^/SF, Dy^+3^/SF, Er^+3^/SF, Tm^+3^/SF, Yb^+3^/SF, and Lu^+3^/SF).

Birefringence results are in agreement with WAXS analyses. In fact, as shown in Figure 6, Ce^+3^/SF, Tm^+3^/SF, Yb^+3^/SF, Lu^+3^/SF, and Pr ^+3^/SF retain the native birefringence of the degummed SF, although with different degrees. This is evidently due to a high degree of crystallinity, with β-sheet nanocrystals highly oriented along the fiber axis, as indicated by WAXS. On the contrary, Dy^+3^/SF, and La^+3^/SF show poor or no birefringence, thus suggesting that regenerated fibers lose or only partially preserve the native crystallinity of degummed SF, with a significative loss in the orientation of the β-sheet domains.

### FTIR-ATR

The secondary structure of Ln^+3^/SF fibers was further characterized by FTIR-ATR spectra. FT-IR spectroscopy enables to elucidate some structural features of proteins since the amide bands are diagnostics of the secondary structure. The amide I band, in the range 1,700–1,600 cm^–1^, mainly originates from the C = O stretching vibrations and minor contributions from the NH in-plane bending, the out of phase CN stretching vibration and the CCN deformations. The amide II band (1,600–1,500 cm^–1^) is the result of C-N stretching and N-H in-plane bending modes whilst the amide III region (1,330–1,230 cm^–1^) arises from N-H in-plane bending vibrations.

**FIGURE 7:**
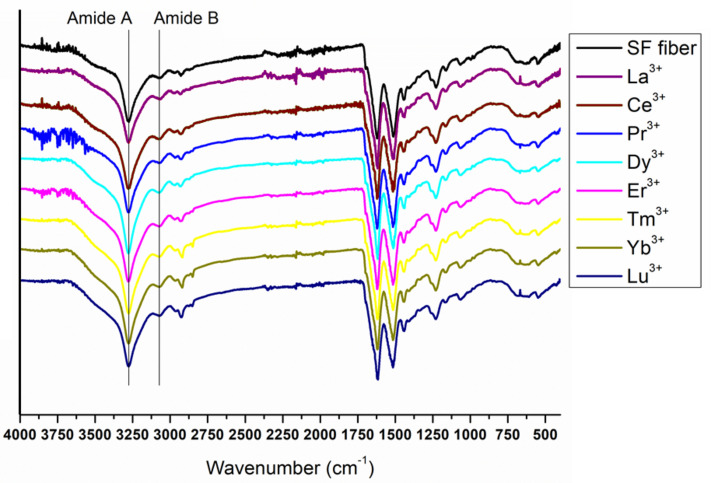
Complete ATR-FTIR spectra of solid degummed SF (black curve) and regenerated solid Ln^+3^/SF (La^+3^/SF, Ce^+3^/SF, Pr^+3^/SF, Dy^+3^/SF, Er^+3^/SF, Tm^+3^/SF, Yb^+3^/SF, and Lu^+3^/SF).

**FIGURE 8:**
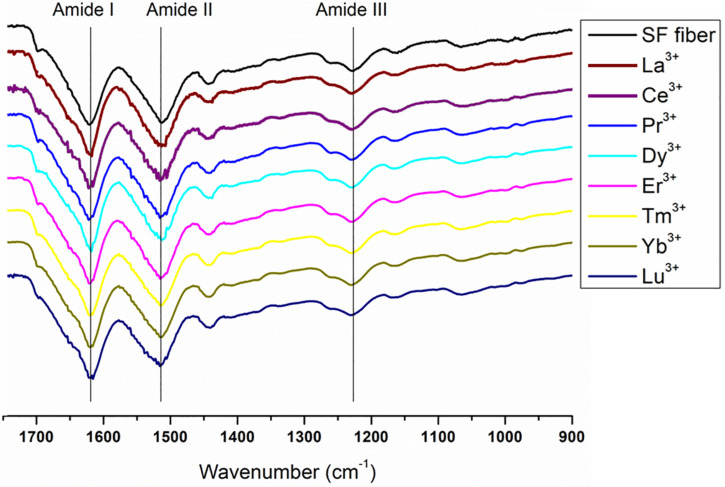
Expansion of the amide band region, between 1750 and 900 cm^− 1^, of the spectra in Figure 7. Solid degummed SF (black curve) and regenerated solid Ln^+3^/SF (La^+3^/SF, Ce^+3^/SF, Pr^+3^/SF, Dy^+3^/SF, Er^+3^/SF, Tm^+3^/SF, Yb^+3^/SF, and Lu^+3^/SF).

The complete spectra of both solid degummed SF and Ln^+3^/SF are reported in Figure 7. The expansions of the amide band region (1,750–900 cm^–1^) are shown in Figure 8.

The ATR-FTIR spectrum of the degummed SF (black curve in Figures 7, 8) shows two strong characteristic absorptions in the amide region at 1,622 cm^–1^ (amide I) and 1,511 cm^–1^ (amide II), and a medium-low intensity band at 1,232 cm^–1^ (amide III), typical of the β-sheet structure, in agreement with the IR spectra reported in the literature (Hu et al., 2006, 2008). Moreover, a very low intensity peak at 1,697 cm^–1^ and broad shoulders centered at 1,668 cm^–1^, that can be assigned to the β-sheet structure and random coil and α-helix regions, respectively, are evident. ATR-FTIR indicated that the β-sheet content is predominant in degummed SF. The ATR-FTIR spectra of SF treated with La^+3^, Ce^+3^, Pr^+3^, Dy^+3^, Er^+3^, Tm^+3^, Yb^+3^, and Lu^+3^ do not show any significant difference compared to the spectrum of the degummed SF, thus suggesting that the treatment with Ln^+3^ ions does not induce any significant variation of the secondary structure of SF.

**FIGURE 9:**
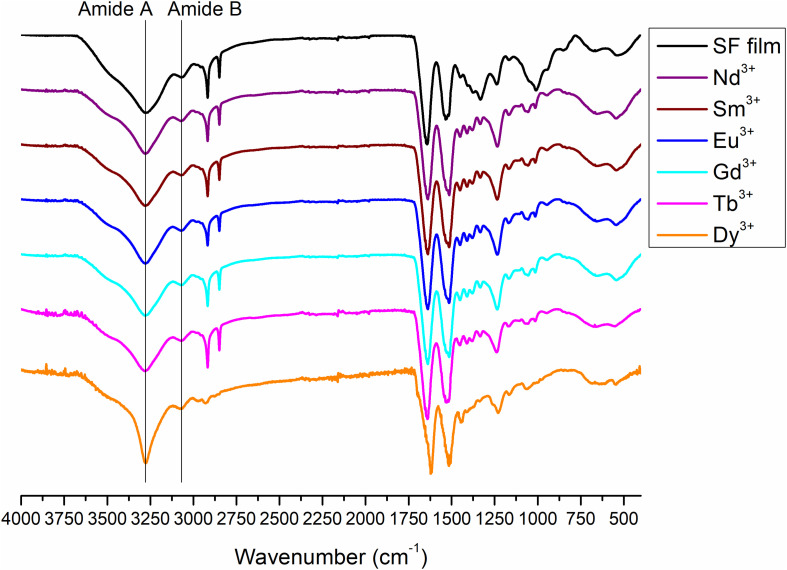
ATR-FTIR spectra of degummed SF film (black curve) and films obtained by drop-casting of solutions of SF with Nd^+3^, Sm^+3^, Eu^+3^, Gd^+3^, Tb^+3^, and Dy^+3^ after purification by dialysis.

**FIGURE 10:**
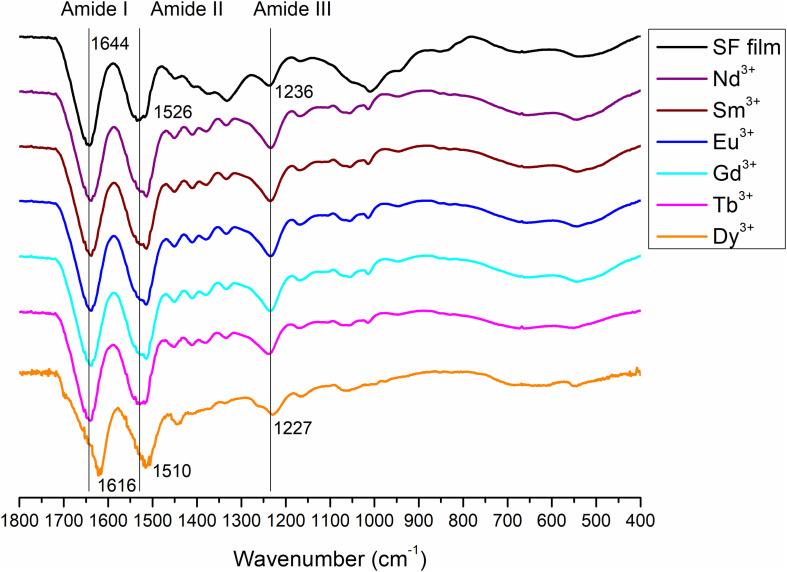
Expansion of the amide band region, between 1,800 and 400 cm^− 1^, of the ATR-FTIR spectra of degummed SF film (black curve) and films obtained by drop-casting of solutions of SF with Nd^+3^, Sm^+3^, Eu^+3^, Gd^+3^,Tb^+3^, and Dy^+3^ after purification by dialysis.

We acquired also ATR-FTIR spectra of drop-casted films obtained from solutions of SF with Nd^+3^, Sm^+3^, Eu^+3^, Gd^+3^, Tb^+3^, and Dy^+3^ (Figures 9, 10) after dialysis. The ATR-FTIR spectrum of the film obtained by drop-casting of SF solution recovered by the standard Ajisawa’s method (black line in Figures 9, 10), i.e., using CaCl_2_ as chaotropic salt, was acquired for comparison.

Typical absorption bands can be identified. In addition to the typical Amide A and B bands (that originate from a Fermi resonance between the first overtone of amide II band and the N-H stretching vibrations) at 3,276 and 3,055 cm^–1^, respectively, the asymmetric and symmetric C-H stretching frequencies at 2,920 and 2,847 cm^–1^ are evident. Amide I, Amide II and Amide III stretching frequencies bands are 1,644, 1,526, and 1,236 cm^–1^. All films obtained from SF solutions with Nd^+3^, Sm^+3^, Eu^+3^, Gd^+3^, Tb^+3^ did not show any appreciable difference in the position and intensity of the typical IR bands. Significant differences are observed only for Dy^+3^/SF, which exhibits an ATR-FTIR spectrum similar to that of the solid fiber with a shift of all the absorptions in the amide region toward lower wave numbers (Amide I 1,616 cm^–1^, Amide II 1,510 cm^–1^, and Amide III at 1,227 cm^–1^). This lowering of the Amide band is strongly indicative of an increase in the β-sheet content in the protein and in the crystallinity of the material.

**FIGURE 11:**
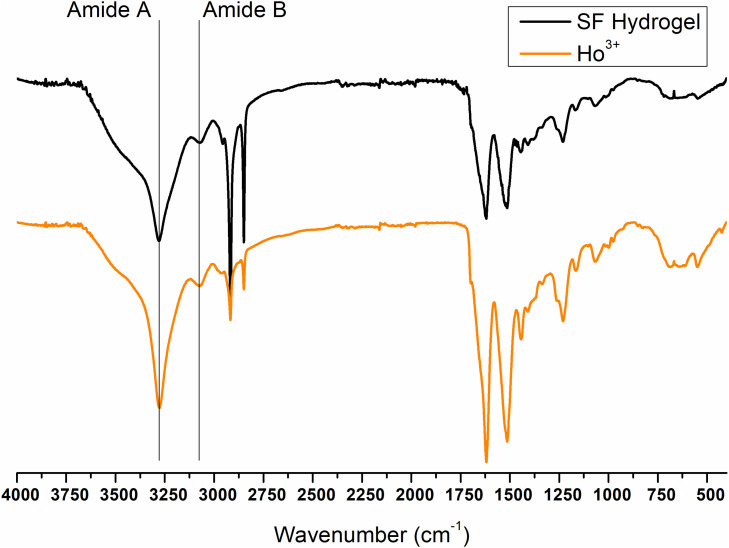
ATR-FTIR spectra of Ho^+3^/SF hydrogel obtained by spontaneous gelation of the dialyzed solution of SF dissolved in HoCl_3_ according to the modified Ajisawa’s protocol.

**FIGURE 12:**
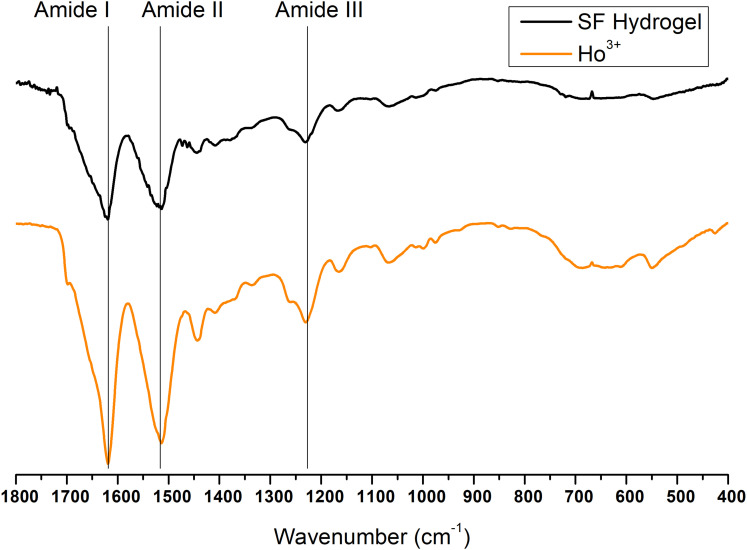
Expansion of the amide band region, between 1,800 and 400 cm^− 1^, of the ATR-FTIR spectra of regenerated Ho^+3^-SF hydrogel.

Finally, the ATR-FTIR spectrum of the SF gel obtained by dissolution with Ho^+3^ is completely superimposable to the spectrum of SF hydrogel obtained by the Ajisawa’s protocol (Figures 11, 12), thus suggesting that Ho^+3^ ions are capable of dissolving SF, as observed for all the lanthanides, but they are not able to regenerate the fibers.

## Discussion

The screening analysis of the behavior of SF solubilized by substituting the chaotropic salt CaCl_2_ with hydrated lanthanides chlorides, LnCl_3_⋅nH_2_O, in the standard Ajisawa’s method led to some very interesting results. First of all, all LnCl_3_⋅nH_2_O are able to solubilize SF when used as chaotropic salts, but lanthanides at the beginning and at the end of the series of the Periodic Table (La^+3^, Ce^+3^, Pr^+3^, Dy^+3^, Er^+3^, Tm^+3^, Yb^+3^, and Lu^+3^) are able to induce the regeneration of the fiber during purification by dialysis. When using intermediate lanthanides (Nd^+3^, Pr^+3^, Sm^+3^, Eu^+3^, Gd^+3^, and Tb^+3^) aqueous solutions of SF are obtained, as in the standard Ajisawa’s method. Dy^+3^ and Ho^+3^ can be considered borderline cases: using Dy^+3^ only 45% of the fiber is recovered during dialysis and the remaining part is in the aqueous solution, Ho^+3^ induces the formation of a SF hydrogel.

The fibers reprecipitated from Pr^+3^, Er^+3^, Tm^+3^, Yb^+3^, and Lu^+3^ solutions in H_2_O/EtOH keep a morphology very similar to that of degummed SF and Ce^+3^/SF: their integrity is almost recovered, although with a reduced smoothness and straightness. The typical hemicylindrical shape of the cross-section of the fibers is also retained, with an increase in the average diameter, probably due to swelling effects. Fibers obtained after treatment with La^+3^ and Dy^+3^ are constituted by small and disordered fragments with very coarse surfaces and irregular cross sections.

WAXS experiments, confirmed by ATR-FTIR and birefringence, demonstrated that in all cases the samples belonging to the SF-fiber regenerating lanthanides group retain an ordered crystalline structure, but they show two different behaviors:

(i) the fibers obtained with Pr^+3^ (at the beginning of the lanthanide series, as Ce^+3^) and Tm^+3^, Yb^+3^, Lu^+3^ (at the end of the series) exhibit 2D WAXS patterns similar to degummed SF and Ce^+3^/SF, with a typical cross β-diffraction pattern, thus indicating that, during reprecipitation, the fibers partially recover a highly oriented structure, rich of stacked β-sheet nanocrystals;

(ii) the fibers obtained with La^+3^, Dy^+3^, and Er^+3^ show 2D WAXS patterns similar to natural silk cocoon, i.e., without any preferential orientation.

Anyway, WAXS experiments show no sign of inclusion of lanthanide ions within the crystalline domains of the fibers, contrary to what was found in the case of Ce^+3^/SF.

XPS characterization indicates that the fibers with La^+3^, Tm^+3^, and Lu^+3^ do not show any relevant contamination of lanthanide ions, whilst in all other fibers the Ln^+3^ concentration is between 0.2 and 0.4% at, but, except for Ce^+3^, Ln^+3^ ions are not coordinated to SF. This evidence could suggest that lanthanide ions could be present as impurities, likely on the fiber surface.

In summary, only when using CeCl_3_⋅7H_2_O as chaotropic salt in the Ajisawa’s method it is possible to obtain SF doped by Ce^+3^ in a significant amount. In all other cases, SF is regenerated, but it is not doped with Ln^+3^ ions and, in some cases, it has different structural properties. Regarding intermediate lanthanide ions, it is interesting to point out that only SF aqueous solutions can be obtained at the end of the Ajisawa’s process and the features of films prepared by solvent evaporation are very similar to the films obtained when using CaCl_2_.

As already stressed when discussing the results obtained in our previous work on Ce^+3^/SF (Rizzo et al., 2020b), this behavior is difficult to rationalize, firstly because silk solubilization and recovery are complex processes, driven by chemical and physical interactions that are not completely understood to date. It is known that SF solubilization is possible only in the presence of chaotropic salts that seemingly disrupt the hydrogen bonding network between the hydration shell of the protein and the bulk, so weakening the hydrophobic effects. However, the roles of different chaotropic salts and especially of solvent molecules have not been clarified yet.

In this context, it is necessary to consider that when using lanthanides as solubilizing agents, the situation is much more complicated due to the particular properties of lanthanides. Lanthanides series consist of 15 elements, from lanthanum (*Z* = 57) to lutetium (*Z* = 71), with high coordination numbers and characteristic oxidation state +3 (Zheng, 2012; Peters et al., 2020). It is well-known that the interactions between Ln^+3^ cations and ligands are mainly of electrostatic origin, since the formation of covalent bonds with donor atoms of ligand is virtually impossible. Due also to the absence of directionality of bonding electrons, the coordination number and the stereochemistry of Ln^+3^-complexes are dictated by the steric requirements of the ligands and not by electronic effects. In addition, the so-called “lanthanide contraction” (Peters et al., 2020), which is a unique characteristic of lanthanide metals, must be taken into account: Ln^+3^ ionic radium decreases with increasing atomic number, that is with increasing charge density. Lanthanide contraction further affects the capability of lanthanide ions to form stable complexes. Ln coordination numbers in the range 3–12 have been observed and with 7, 8, and 9 as the most common ones. Lanthanides ions with larger ionic radii can, in principle, have larger coordination numbers. On the other hand, the decrease of ionic radium with the consequent increase of charge density increases the tendency to form more stable complexes. In any case, due to their electrostatic nature, Ln^+3^-complexes are quite labile and the coordination sphere is not well-defined, since the coordination number is primarily affected by the size of the ligands, the number of donors of each ligand that can pack around the ion and, in the case of bulky ligands, by intermolecular interactions. Because of the small radius and the high nuclear charge, the Ln^+3^ ions are strong Lewis acids that coordinate with hard bases such as carboxylates and highly electronegative donors such as N or O (Zheng, 2012), that is to say that Ln^+3^ ions are good complexing agents for amino acids and peptide bonds in proteins.

In our previous paper (Rizzo et al., 2020b), comparing the behavior of SF in the presence of Ce^+3^ ions to the results reported in the literature on the influence of other metal ions on SF, it seemed reasonable to infer that an important role is played by Ce^+3^ charge and coordination number. The results obtained for the complete series of lanthanides are consistent with this hypothesis.

The behavior of SF when Ln^+3^ ions in the middle of the lanthanide series (Nd^+3^, Pr^+3^, Sm^+3^, Eu^+3^, Gd^+3^, and Tb^+3^) are used as chaotropic salts is similar to the behavior observed in the presence of Ca^+2^ ions (standard Ajisawa’s method). This can be explained as an effect of the ionic radius. In fact, as it can be seen in Table 1, the ionic radii of Ln^+3^ from Nd^+3^ to Tb^+3^, are within 1.08 and 1.01 Å, comparable to the ionic radius of Ca^+2^ (1.03 Å).

For lanthanides at the beginning and at the end of the series, the Ln^+3^ coordination number seems to play a key role, besides the ionic radius. During the purification step after solubilization, the removal of Ln^+3^ ions, as already found for Ce^+3^, seems to promote the self-assembly of the fiber in the transition from solution to solid state, with the fibers undergoing a structural transition from random coil to a highly ordered structure.

Ce^+3^ ion has ionic radius slightly larger than that of Ca^+2^, but also a larger charge and coordination number. Pr^+3^ has an ionic radius slightly lower than Ce^+3^, and presumably a lower coordination number. Hence, the fibers obtained at the end of the purification process are very similar to Ce^+3^/SF, although not doped by Pr^+3^ ions, that are more easily removed during the dialysis step, due to its lower coordination number.

Ln^+3^ ions at the end of the series, from Er^+3^ to Lu^+3^, have ionic radii that are significantly lower than Ce^+3^. Even if their density charges are larger than Ce^+3^, their coordination number is smaller, considering that the steric hindrance of the environment is the same. This could imply that these ions can easily induce the solubilization of SF with a mechanism very similar to the one hypothesized for Ce^+3^ ions, but can also been removed more easily than Ce^+3^ ions without remaining trapped within the fiber. The SF reprecipitated after dialysis is highly ordered, and richer of stacked β-sheet nano-crystals as the ionic radius decreases.

## Conclusion

We have reported the results of a systematic investigation on the behavior of degummed SF when solubilized by replacing CaCl_2_ with LnCl_3_⋅nH_2_O in the standard Ajisawa’s reagent. This study builds on previous work using CeCl_3_⋅7H_2_O (Rizzo et al., 2020b) and extends to all the other lanthanides in the Periodic Table, given the potential that lanthanide doping of SF holds for the manufacturing of devices for photonics and electronics.

It would seem that Cerium constitutes a unique case for obtaining lanthanide doped SF. Indeed, using CeCl_3_ as chaotropic salt in water and ethanol, not only it is possible to regenerate SF in a fibrous form, but the fiber obtained is doped with Ce^+3^ ions and preserves the morphological and molecular structure of the pristine SF. Ce^+3^ ions should be coordinated to SF preferentially by interactions with the oxygen atoms of protein backbone and should be distributed within the highly ordered hydrophobic β-sheet regions of SF, as well as amorphous regions (Rizzo et al., 2020b). For all the other re-precipitated fibers (with the exception of La^+3^/SF, Tm^+3^/SF, and Lu^+3^/SF), a surface concentration of Ln^+3^ between 0.2 and 0.4% at was measured, comparable to that measured for Ce^+3^/SF. However, contrary to what was found in the case of Ce^+3^/SF, there is no evidence that the ions are coordinated with the fiber fibroin, nor is the presence of Ln^+3^ ions detectable within the ordered domains. This could suggest that Ln^+3^ ions could be distributed on the fiber surface, although it is not possible to exclude their inclusion within the amorphous regions.

However, the results obtained for the other lanthanides can be considered interesting for other reasons. Indeed, our work shows that all lanthanides in H_2_O/EtOH (8:1) are effective chaotropic salts for SF and that they constitute a valid alternative to CaCl_2_ in the Ajisawa’s method. Using hydrated LnCl_3_ we have obtained a new protocol which enables to regenerate SF in the form of fibrous material by direct reprecipitation during dialysis.

A further appealing feature of the protocol described is its environmental sustainability, given both the possibility of near-full recovery of the lanthanide salts from water and the absence of toxic solvents or hazardous reagents throughout the whole process (Rizzo et al., 2020b).

Further studies are in progress to investigate the optical properties and applications of the fibers regenerated in the presence of lanthanide solutions and also to optimize possible protocols for a controlled inclusion of lanthanide ions other than Cerium into the fibers.

## Data Availability Statement

The original contributions presented in the study are included in the article/Supplementary Material, further inquiries can be directed to the corresponding author/s.

## Author Contributions

All authors listed have made a substantial, direct and intellectual contribution to the work, and approved it for publication.

## Conflict of Interest

The authors declare that the research was conducted in the absence of any commercial or financial relationships that could be construed as a potential conflict of interest.

## References

[B1] Ajisawa A. (1998). Dissolution aqueous of silk fibroin with calcium chloride/ethanol solution. *J. Sericult. Sci. Japan* 67 91–94.

[B2] Altamura D. Lassandro R. Vittoria F. A. De Caro L. Siliqi D. Ladisa M. (2012). X-ray microimaging laboratory (XMI-LAB). *J. Appl. Cryst.* 45 869–873. 10.1107/s0021889812025733

[B3] Chahal R. Starecki F. Boussard-Pledel C. Doualan J.-L. Michel K. Brillard L. (2016). Fiber evanescent wave spectroscopy based on IR fluorescent chalcogenide fibers. *Sensors Act. B* 229 209–216. 10.1016/j.snb.2016.01.091

[B4] da Rocha E. G. Pugina R. S. Caiut J. M. A. (2020). Luminescent sensor based on the lanthanide-fibroin composite. *Opt. Mater.* 109 110236–110244. 10.1016/j.optmat.2020.110236

[B5] Digonnet M. J. (2001). *Rare-Earth-Doped Fiber Lasers and Amplifiers, Revised and Expanded.* Boca Raton, FL: CRC press.

[B6] Fossey S. A. Némethy G. Gibson K. D. Scheraga H. A. (1991). Conformational energy studies of β-sheets of model silk fibroin peptides. I. Sheets of poly(Ala-Gly) chains. *Biopolymers* 31, 1529–1541. 10.1002/bip.360311309 1814502

[B7] Founda I. M. El-Tonsy M. M. (1990). Birefringence behavior of annealed silk fibres. *J. Mater. Sci.* 25 4752–4757. 10.1007/bf01129936

[B8] Georgakoudi I. Tsai I. Greiner C. Wong C. DeFelice J. Kaplan D. (2007). Intrinsic fluorescence changes associated with the conformational state of silk fibroin in biomaterial matrices. *Opt Express* 15 1043–1053. 10.1364/oe.15.001043 19532332

[B9] Guo C. Li C. Mu X. Kaplan D. L. (2020). Engineering silk materials: from natural spinning to artificial processing. *Appl. Phys. Rev.* 7:011313. 10.1063/1.5091442 PMC8340942 34367402

[B10] Guo C. Zhang J. Jordan J. S. Wang X. Henning R. W. Yarger J. L. (2018). Structural comparison of various silkworm silks: an insight into the structure-property relationship. *Biomacromolecules* 19 906–917. 10.1021/acs.biomac.7b01687 29425447

[B11] Guo X. Ge M. Zhao J. (2011). Photochromic properties of rare-earth strontium aluminate luminescent fiber. *Fibers Polym.* 12 875–879. 10.1007/s12221-011-0875-9

[B12] Hu X. Kaplan D. Cebe P. (2006). Determining beta-sheet crystallinity in fibrous proteins by thermal analysis and Infrared Spectroscopy. *Macromolecules* 39 6161–6170. 10.1021/ma0610109

[B13] Hu X. Kaplan D. Cebe P. (2008). Dynamic protein-water relationships during β-sheet formation. *Macromolecules* 41 3939–3948. 10.1021/ma071551d

[B14] Huang W. Ling S. Li C. Omenetto F. G. Kaplan D. L. (2018). Silkworm silk-based materials and devices generated using bio-nanotechnology. *Chem. Soc. Rev.* 47 6486–6504. 10.1039/c8cs00187a 29938722 PMC6113080

[B15] Koh L. D. Cheng Y. Teng C. P. Khin Y. W. Loh X. J. Tee S. Y. (2015). Structures, mechanical properties and applications of silk fibroin materials. *Prog. Polym. Sci.* 46 86–110.

[B16] Kundu B. Rajkhowa R. Kundu S. C. Wang X. (2013). Silk fibroin biomaterials for tissue regenerations. *Adv. Drug Deliv. Rev.* 65 457–470. 10.1016/j.addr.2012.09.043 23137786

[B17] Lawrence B. D. Omenetto F. Chui K. Kaplan D. L. (2008). Processing methods to control silk fibroin film biomaterial features. *J. Mater. Sci.* 43 6967–6985. 10.1007/s10853-008-2961-y

[B18] Lee O. J. Sultan M. D. T. Hong H. Lee Y. J. Lee J. S. Lee H. (2020). Recent Advances in Fluorescent Silk Fibroin. *Front. Mater.* 7:50. 10.3389/fmats.2020.00050

[B19] Ling S. Kaplan D. L. Buehler M. J. (2018). Nanofibrils in nature and materials engineering. *Nat. Rev. Mater.* 3:18016. 10.1038/natrevmats.2018.16 PMC8221570 34168896

[B20] Liu J. Xu J. Guo X. Liao T. Huang Y. (2020). The up-conversion luminescence in the Er^+3^/Yb^+3^/Tm^+3^ tri-doped tellurite glass microsphere coupled by the tapered fiber. *Opt. Sens. Imag. Technol.* 11567 1156740–1156759.

[B21] Marsh R. E. Corey R. B. Pauling L. (1955). An investigation of the structure of silk fibroin. *Bioch. Biophys. Acta* 16 1–34. 10.1016/0006-3002(55)90178-5 14363226

[B22] Melke J. Midha S. Ghosh S. Ito K. Hofmann S. (2016). Silk Fibroin as biomaterial for bone tissue engineering. *Acta Biomater.* 31 1–16. 10.1016/j.actbio.2015.09.005 26360593

[B23] Moulder F. Stickle W. F. Sobol P. E. Bomben K. E. (1992). *Handbook of X-ray Photoelectron Spectroscopy*, ed. Chastain J. (Eden Prairie, MN: Perkin-Elmer Corporation).

[B24] Omenetto F. G. Kaplan D. L. (2010). New opportunities for an ancient material. *Science* 329 528–531. 10.1126/science.1188936 20671180 PMC3136811

[B25] Peters J. A. Djanashvili K. Geraldes C. F. G. C. Platas-Iglesias C. (2020). The chemical consequences of the gradual decrease of the ionic radius along the Ln-series. *Coord. Chem. Rev.* 406:213146. 10.1016/j.ccr.2019.213146

[B26] Pugina R. S. da Rocha E. G. Caiut J. M. A. (2019a). Beta-diketones in the intensification of the luminescence of the silk fibroin films doped rare earth ions. *J. Mater. Sci.: Mater. Electron.* 30 16732–16739. 10.1007/s10854-019-01181-8

[B27] Pugina R. S. da Rocha E. G. Ribeiro S. J. L. Caiut J. M. A. (2019b). Study of the energy transfer process in rare earth-doped silk fibroin for future application in luminescent compounds. *J. Lumin.* 205 423–428. 10.1016/j.jlumin.2018.09.050

[B28] Qi Y. Wang H. Wei K. Yang Y. Zheng R.-Y. Kim I. S. (2017). A review of structure construction of silk fibroin biomaterials form single structures to multi-level structures. *Int. J. Mol. Sci.* 18 237–257. 10.3390/ijms18030237 28273799 PMC5372488

[B29] Reddy N. (2020). *Silk: Materials, Processes, and Applications.* Sawston: Woodhead Publishing.

[B30] Rizzo G. Albano G. Lo Presti M. Milella A. Omenetto F. G. Farinola G. M. (2020a). Palladium supported on silk fibroin for Suzuki-Miyaura cross-coupling reactions. *Eur. J. Org. Chem.* 2020 6992–6996. 10.1002/ejoc.202001120

[B31] Rizzo G. Lo Presti M. Giannini C. Sibillano T. Milella A. Matzeu G. (2020b). Silk fibroin processing from CeCl_3_ aqueous solution: fibers regeneration and doping with Ce(III). *Macromol. Chem. Phys.* 221:2000066. 10.1002/macp.202000066

[B32] Rockwood D. N. Preda R. C. Yücel T. Wang X. Lovett M. L. Kaplan D. L. (2011). Materials fabrication from Bombyx mori silk fibroin. *Nat. Protoc.* 6 1612–1631. 10.1038/nprot.2011.379 21959241 PMC3808976

[B33] Seaborg G. T. (1978). “Actinides and Transactinides,” in *Kirk-Othmer: Encyclopedia of Chemical Technology*, Vol. 1 ed. Othmer D. (New York, NY: John Wiley and Sons), 456–488.

[B34] Shen X. Hu Q. Ge M. (2021). Fabrication and characterization of multi stimuli-responsive fibers via wet-spinning process. *Spectrochim. Acta Part A Mol. Biomol. Spectrosc.* 250 119245. 10.1016/j.saa.2020.119245 33303381

[B35] Shen Y. Johnson M. A. Martin D. C. (1998). Microstructural characterization of *Bombyx mori* silk fibers. *Macromolecules* 31 8857–8864.

[B36] Sibillano T. De Caro L. Scattarella F. Scarcelli G. Siliqi D. Altamura D. (2016). Interfibrillar packing of bovine cornea by table-top and synchrotron scanning SAXS microscopy. *J. Appl. Cryst.* 49 1231–1239. 27504077 10.1107/S1600576716010396 PMC4970496

[B37] Siliqi D. De Caro L. Ladisa M. Scattarella M. Mazzone A. Altamura D. (2016). SUNBIM: a package for X. ray imaging of nano-and biomaterials using SAXS, WAXS, GISAXS, GIWAXS techniques. *J. Appl. Cryst.* 49 1107–1114.

[B38] Tao H. Kaplan D. L. Omenetto F. G. (2012). Silk materials – A road to sustainable high technology. *Adv. Mater.* 24 2824–2837. 22553118 10.1002/adma.201104477

[B39] Viney C. Huber A. E. Dunaway D. L. Kerkam K. Case S. T. (1993). “Optical characterization of silk secretions and fibers,” in *Silk Polymers: Material Science and Biotechnology*, eds Kaplan D. Adams W. W. Farmer B. Viney C. (Washington, D.C: ACS Publication), 120–136.

[B40] Zheng X. Zhao M. Zhang H. Fan S. Shao H. Hu X. (2018). Intrinsically fluorescent silks from silkworms fed with rare-earth upconverting phosphors. *ACS Biomater. Sci. Eng.* 4 4021–4027. 33418802 10.1021/acsbiomaterials.8b00986

[B41] Zheng Z. (2012). *Lanthanides: Amino Acid Compounds. Encyclopedia of Inorganic and Bioinorganic Chemistry, Online.* Hoboken, NJ: John Wiley & Sons, Ltd,

[B42] Zhu B. Wang H. Leow W. R. Cai Y. Loh X. J. Han M.-Y. (2016). Silk fibroin for flexible electronic devices. *Adv. Mater.* 28 4250–4265. 26684370 10.1002/adma.201504276

