# Supplementary material for: Bombyx mori Silk Fibroin Regeneration in Solution of Lanthanide Ions: A Systematic Investigation
Source: Front Bioeng Biotechnol. 2021 Jun 10;9:653033. doi: 10.3389/fbioe.2021.653033 (PMC8222627; doi:10.3389/fbioe.2021.653033)
Supplement: Supplementary file 1 [file Data_Sheet_1.PDF]

Supporting Information

Bombyx mori silk fibroin regeneration in solution of lanthanide ions: a systematic investigation

Giorgio Rizzo[1], Marco Lo Presti[2], Cinzia Giannini[3], Teresa Sibillano[3], Antonella Milella[1], Giulia Guidetti[2], Roberta Musio[1]*, Fiorenzo G. Omenetto[2]* and Gianluca M. Farinola[1,2]*

[1]Dipartimento di Chimica, Università degli Studi di Bari “Aldo Moro”, Bari, Italy
[2]Silklab, Department of Biomedical Engineering, Tufts University, Medford, Massachusetts, USA
[3]CNR IC - Institute of Crystallography, Bari, Italy

CONTENTS

1. Lanthanide salts used for SF dissolution/regeneration protocol page 2
2. SEM images of Ln^{+3}/SF fibers page 3
3. Quantitative elemental composition of Ln^{+3}/SF fibers page 5
4. 2D WAXS pattern analysis page 6

Table S1. Quantity of lanthanide salts used for SF dissolution/regeneration protocol. For comparison, calcium chloride standard salt is listed in the last row.

Lanthanide (or Lanthanoid)	Salt	Salt quantity (g)	Result
Lanthanum	$LaCl_3 \cdot 7H_2O$	1.766	Fibers
Cerium	$CeCl_3 \cdot 7H_2O$	1.772	Fibers
Praseodymium	$PrCl_3 \cdot 7H_2O$	1.777	Fibers
Neodymium	$NdCl_3 \cdot 6H_2O$	1.705	Solution
Samarium	$SmCl_3 \cdot 6H_2O$	1.736	Solution
Europium	$EuCl_3 \cdot 6H_2O$	1.744	Solution
Gadolinium	$GdCl_3 \cdot 6H_2O$	1.768	Solution
Terbium	$TeCl_3 \cdot 6H_2O$	1.776	Solution
Dysprosium	$DyCl_3 \cdot 6H_2O$	1.792	Solution/Fibers
Holmium	$HoCl_3 \cdot 6H_2O$	1.804	Gel
Erbium	$ErCl_3 \cdot 6H_2O$	1.815	Fibers
Thulium	$TmCl_3 \cdot 6H_2O$	1.825	Fibers
Ytterbium	$YbCl_3 \cdot 6H_2O$	1.844	Fibers
Lutetium	$LuCl_3 \cdot 6H_2O$	1.854	Fibers
Calcium	$CaCl_2$	0.528	Solution

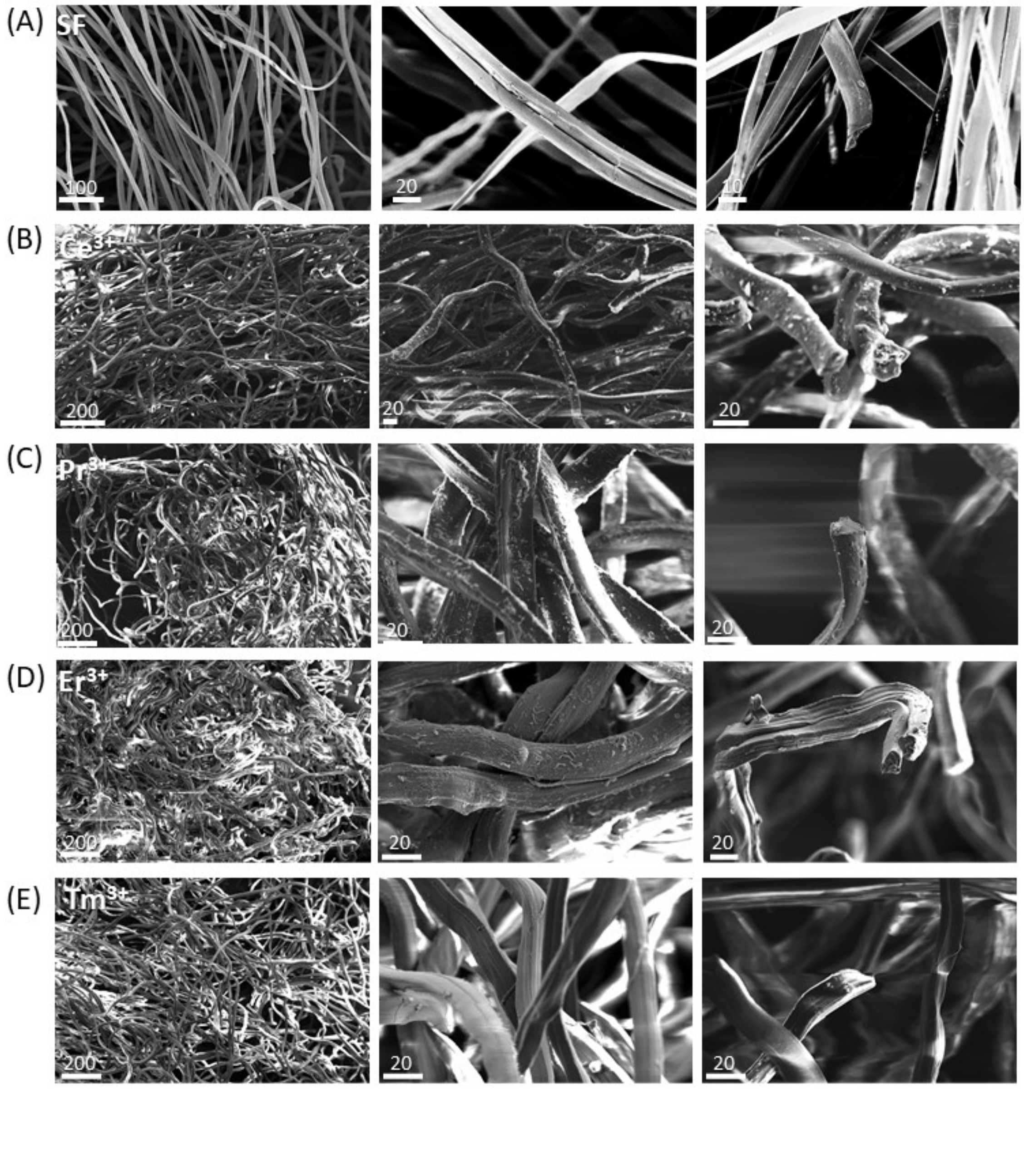

Figure S1(part A). SEM images of regenerated Ln^{+3}/SF fibers

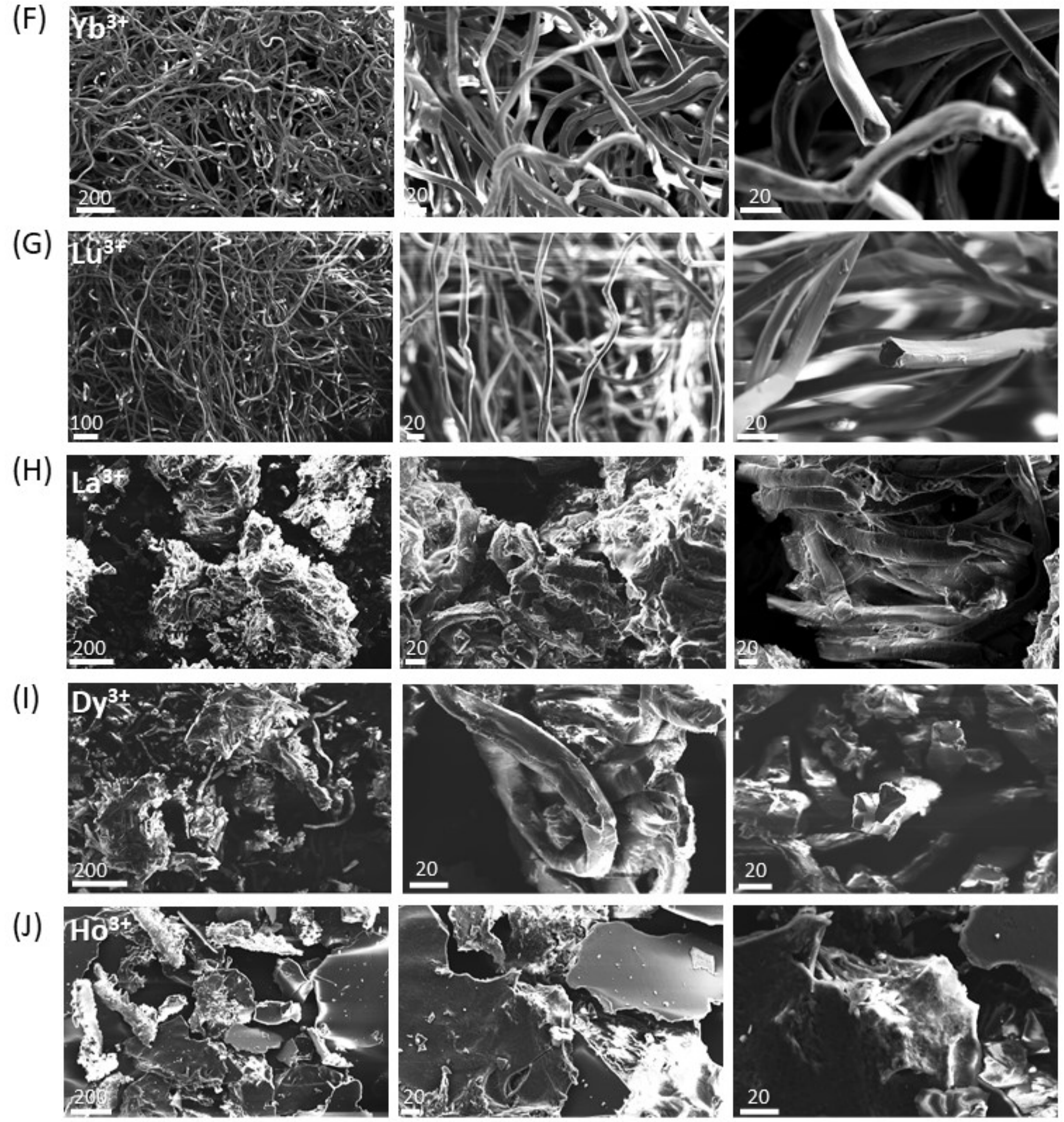

Figure S1(part B). SEM images of regenerated Ln^{+3}/SF fibers

Table S2. XPS elemental composition of Ln^{+3}/SF regenerated fibers.

Sample	Peak	Ln at%	C1s at%	O1s at%	N1s at%
La^{+3}/SF	La3d	<0.1	83.1	16.4	0.5
Ce^{+3}/SF	Ce3d	0.3	68.6	19.5	11.6
Pr^{+3}/SF	Pr3d	0.4	62.7	22.9	14.0
Dy^{+3}/SF	Dy4d	0.3	83.9	14.9	0.9
Er^{+3}/SF	Er4d	0.2	63.8	21.9	14.1
Tm^{+3}/SF	Tm4d	<0.1	75.4	15.3	9.3
Yb^{+3}/SF	Yb4d	0.3	80.8	18.6	0.3
Lu^{+3}/SF	Lu4f	<0.1	66.0	21.5	12.5

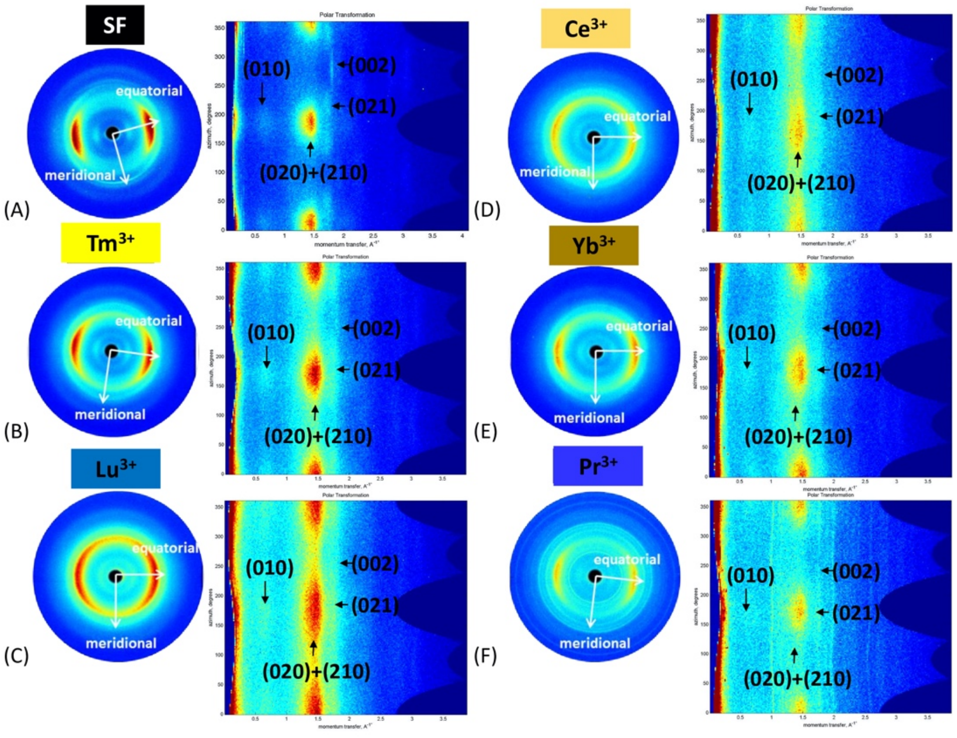

Figure S2. 2D WAXS patterns of (a) silk fibroin; (b) Ce^{3+}-SF; (c) Tm^{3+}/SF; (d) Yb^{3+}/SF; (e) Lu^{3+}/SF; (f) Pr^{3+}/SF with the corresponding polar transformation.

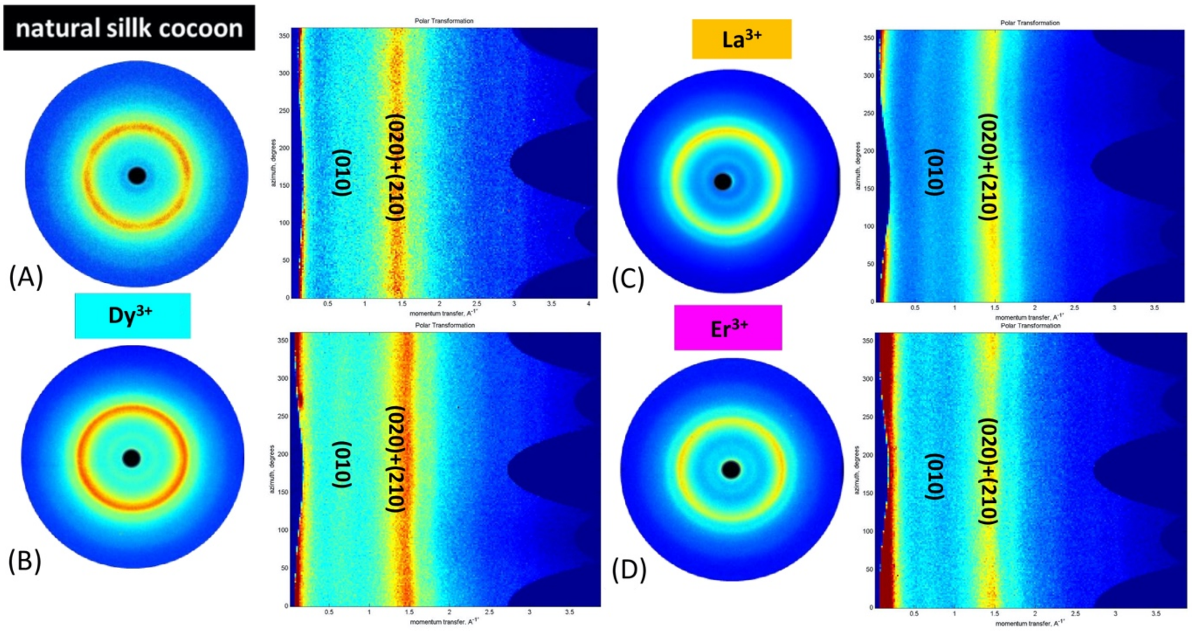

Figure S3. 2D WAXS patterns of (a) natural silk cocoon; (b) La^{+3}/SF; (c) Dy^{+3}/SF; (d) Er^{+3}/SF with the corresponding polar transformation.

Table S3. d-spacings and corresponding q values, calculated for *Bombyx mori* silk II structure (orthorhombic structure with unit cell dimensions a= 9.68 ±0.20 Å, b= 9.36± 0.18 Å and c=7.02± 0.14 Å).

hkl	**d-spacing (Å) (calculated)**	**q ($Å^{-1}$) (calculated)**
010	9.36	0.67
020	4.68	1.34
030	3.12	2.01
110	6.72	0.93
210	4.3	1.46
310	3.05	2.06
011	5.61	1.12
021	3.89	1.61
031	2.851	2.20
111	4.85	1.29
211	3.66	1.72
311	2.79	2.25
001	7.02	0.89
002	3.51	1.79
